# Trends in maternal prepregnancy body mass index (BMI) and its association with birth and maternal outcomes in California, 2007–2016: A retrospective cohort study

**DOI:** 10.1371/journal.pone.0222458

**Published:** 2019-09-19

**Authors:** Anura W. G. Ratnasiri, Henry C. Lee, Satyan Lakshminrusimha, Steven S. Parry, Vivi N. Arief, Ian H. DeLacy, Jo-Shing Yang, Ralph J. DiLibero, Julia Logan, Kaye E. Basford

**Affiliations:** 1 Benefits Division, California Department of Health Care Services, Sacramento, California, United States of America; 2 School of Agriculture and Food Sciences, Faculty of Science, The University of Queensland, Brisbane, Queensland, Australia; 3 Division of Neonatology, School of Medicine, Stanford University, Stanford, California, United States of America; 4 Department of Pediatrics, School of Medicine, University of California Davis, Sacramento, California, United States of America; 5 Anesthesia Room ML, University of California San Francisco, San Francisco, California, United States of America; 6 Health Plan Administration Division, California Public Employees' Retirement System, Sacramento, California, United States of America; 7 School of Biomedical Sciences, Faculty of Medicine, The University of Queensland, Brisbane, Queensland, Australia; University of Botswana, BOTSWANA

## Abstract

**Objective:**

To determine recent trends in maternal prepregnancy body mass index (BMI) and to quantify its association with birth and maternal outcomes.

**Methods:**

A population-based retrospective cohort study included resident women with singleton births in the California Birth Statistical Master Files (BSMF) database from 2007 to 2016. There were 4,621,082 women included out of 5,054,968 women registered in the database. 433,886 (8.6%) women were excluded due to invalid or missing information for BMI. Exposures were underweight (BMI < 18.5 kg/m^2^), normal weight (18.5–24.9 kg/m^2^), overweight (25.0–29.9 kg/m^2^), and obese (≥ 30 kg/m^2^) at the onset of pregnancy. Obesity was subcategorized into class I (30.0–34.9 kg/m^2^), class II (35.0–39.9 kg/m^2^), and class III (≥ 40 kg/m^2^), while adverse outcomes examined were low birth weight (LBW), very low birth weight (VLBW), macrosomic births, preterm birth (PTB), very preterm birth (VPTB), small-for-gestational-age birth (SGA), large-for-gestational-age birth (LGA), and cesarean delivery (CD). Descriptive analysis, simple linear regression, and multivariate logistic regression were performed, and adjusted odds ratios (AORs) with 95% confidence intervals (CIs) for associations were estimated.

**Results:**

Over the ten-year study period, the prevalence of underweight and normal weight women at time of birth declined by 10.6% and 9.7%, respectively, while the prevalence of overweight and obese increased by 4.3% and 22.9%, respectively. VLBW increased significantly with increasing BMI, by 24% in overweight women and by 76% in women with class III obesity from 2007 to 2016. Women with class III obesity also had a significant increase in macrosomic birth (170%) and were more likely to deliver PTB (33%), VPTB (66%), LGA (231%), and CD (208%) than women with a normal BMI. However, obese women were less likely to have SGA infants; underweight women were 51% more likely to have SGA infants than women with a normal BMI.

**Conclusions:**

In California from 2007 to 2016, there was a declining trend in women with prepregnancy normal weight, and a rising trend in overweight and obese women, particularly obesity class III. Both extremes of prepregnancy BMI were associated with an increased incidence of adverse neonatal outcomes; however, the worse outcomes were prominent in those women classified as obese.

## Introduction

The worldwide obesity epidemic continues to be a major public health challenge, particularly in women of childbearing age [1–3]. Obesity is now defined using the World Health Organization (WHO) criteria based on body mass index (BMI), which is calculated by dividing the weight in kilograms by the square of height in meters [4]. Women who become pregnant while their BMI is below or above the normal range (18.5 to 24.9 kg/m^2^) are now known to have an increased risk of adverse maternal pregnancy outcomes and adverse birth outcomes [5–6].

A national survey of adults in the United States showed that the age-adjusted incidence of obesity (BMI ≥ 30 kg/m^2^) in women was 40.4% in 2013 to 2014 [7]. The corresponding value for class 3 obesity (BMI ≥ 40 kg/m^2^) in women was 9.9% [7]. The prevalence of both overall obesity and class 3 obesity showed a significant linear increase from 2005 to 2014 [7]. In 2014, half of pregnant women were either overweight (25.6%) or obese (24.8%) [8].

Prepregnancy obesity affects the health of both mother and child [9]. Maternal complications associated with obesity during pregnancy include an increased risk of cesarean delivery, miscarriage, pre-eclampsia, gestational diabetes, and thromboembolism [9–12]. Obesity during pregnancy has adverse consequences for placental, embryonic, and fetal growth; increases infant mortality; and increases the risk for postpartum complications [9,10,13–15].

Experimental and clinical evidence suggest that maternal obesity also has long-lasting consequences for the health of the offspring [9]. Maternal obesity is associated with an increased BMI in children during infancy [16], adolescence [17], and into adulthood [18]. Maternal obesity during gestation is also linked with an increased risk for coronary heart disease, diabetes mellitus, stroke, asthma, and premature death in adult offspring [9,19–21]. Maternal obesity is a serious public health problem that has both immediate and long-term consequences. It has been associated with increased use of healthcare services, including longer hospital stays during pregnancy [22]. Because of the sheer number of infants born to an ever-increasing number of women with prepregnancy obesity, there is a need to understand the associations between maternal obesity and birth outcomes.

Few studies have focused on recent trends in maternal prepregnancy BMI and birth and maternal outcomes. Our aim was to determine temporal trends and patterns in prepregnancy BMI and to quantify its associations with birth and maternal outcomes in California over a 10-year period. The adverse outcomes evaluated were low birth weight (LBW), very low birth weight (VLBW), macrosomic birth, preterm birth (PTB), very preterm birth (VPTB), small-for-gestational-age (SGA), large-for-gestational-age (LGA), and cesarean delivery (CD).

## Materials and methods

### Data source

We conducted a population based retrospective cohort study by analyzing the California Birth Statistical Master Files (BSMF) compiled by the Center for Health Statistics and Informatics, California Department of Public Health (CDPH), which recorded all births in California for the 10-year period from 2007 to 2016. Recording of self-reported maternal prepregnancy weight and height in the birth records commenced in California during 2007 allowing for the calculation of maternal prepregnancy BMI [4].

We excluded records with missing data on maternal weight or height, or out-of-range values [23]. Further data screening was performed to include all live births, delivered in the range of 17 to 47 completed weeks of gestation based on the best obstetric estimate, with a birth weight of 500 grams or greater [24–25].

This study was approved by the California Committee for the Protection of Human Subjects (CPHS Protocol ID: 16-10-2759) and the CDPH Vital Statistics Advisory Committee.

#### Outcome variables: LBW, VLBW, macrosomic birth, PTB, VPTB, SGA, LGA, and cesarean delivery

Birth weight data obtained from the BSMF were coded as dichotomous variables, indicating whether the infant was LBW (< 2500 g) or not; or VLBW (< 1500 g) or not. Both PTB and VPTB were used as outcome variables. A macrosomic birth was defined as a birth weight greater than 4500 grams; this was also coded as a dichotomous variable. Data on PTB and VPTB were coded as dichotomous variables, indicating whether the infant underwent PTB (< 37 weeks gestational age) or not; and whether the infant underwent VPTB (< 32 weeks gestational age) or not. We grouped birth weight and gestational age into three groups as described by Ratnasiri et al. (2018) [26] using new gender specific intrauterine growth curves based on United States data by Olsen et al. (2010) [27]: small-for-gestational-age (SGA) (< 10th percentile), appropriate-for-gestational-age (AGA) (10th to 90th percentile), and large-for-gestational-age (LGA) (> 90th percentile). SGA, LGA, and cesarean delivery (CD) were categorized as dichotomous variables as described above.

#### Main exposure

**Fig 1:**
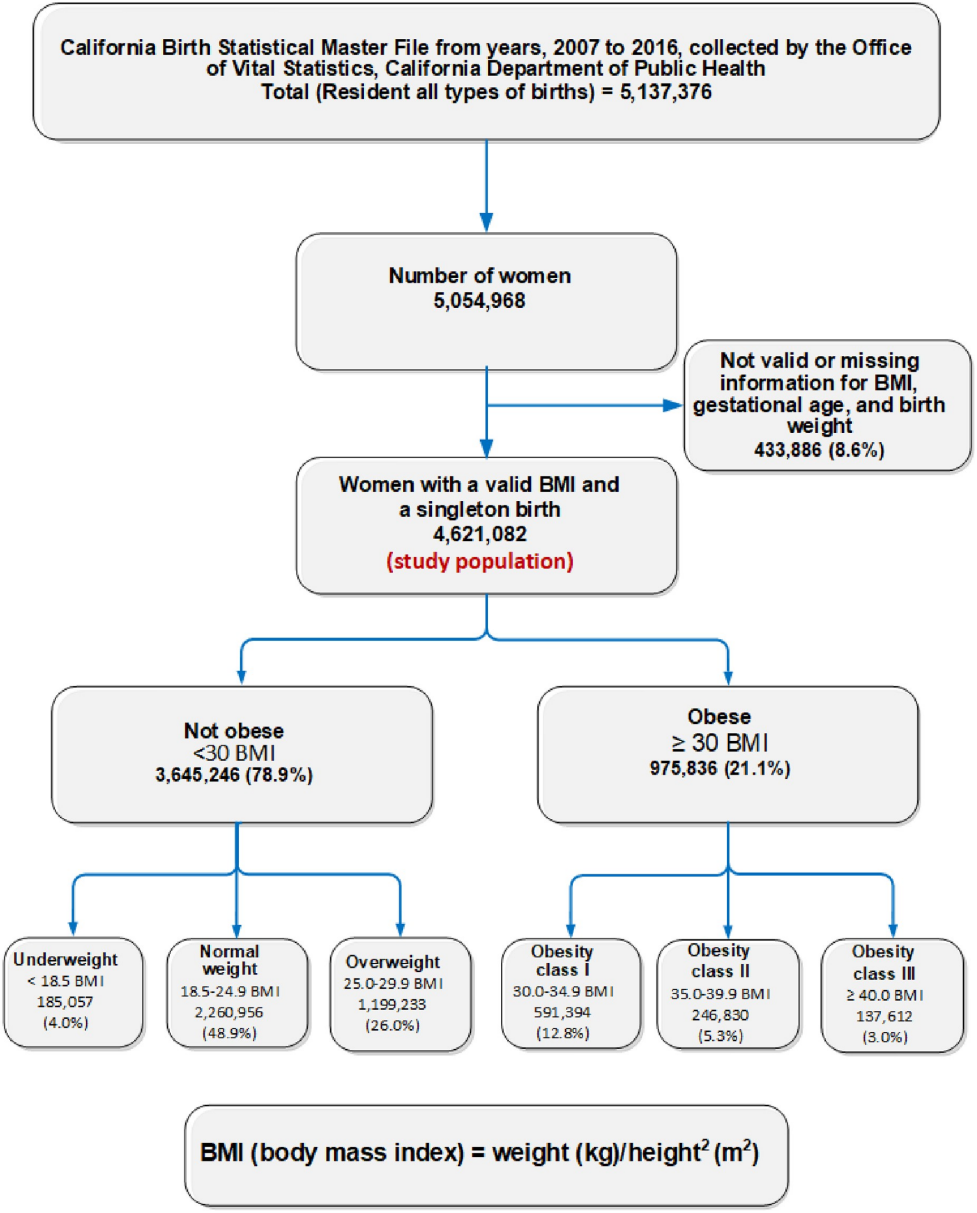
Screening criteria to identify the study population of eligible women from the California Birth Statistical Master File for the period 2007–2016.

Using the WHO criteria, prepregnancy BMI was divided into the following categories (Fig 1): underweight, less than 18.5kg/m^2^; normal, 18.5 to 24.9 kg/m^2^; overweight, 25.0 to 29.9 kg/m^2^; obesity class I, 30.0 to 34.9 kg/m^2^; obesity class II, 35.0 to 39.9 kg/m^2^; and obesity class III, 40 kg/m^2^ or greater (4) and used maternal prepregnancy BMI category as the main exposure.

#### Covariates

The covariates identified were birth year, maternal sociodemographic status, prenatal smoking, type of health insurance, parity, and use of prenatal care [28]. Maternal sociodemographic status included maternal age, education level, race and ethnicity, maternal nativity, and geographic region. The health insurance types considered were Medi-Cal (public) and private insurance. Data were also available on whether or not the women in this study were part of the Federal Supplemental Nutrition Program for Women, Infants, and Children (WIC). Having Medi-Cal insurance or being part of the WIC program were considered predictive of low incomes.

The perinatal health behavioral characteristics included maternal smoking during pregnancy. The birth records included self-reported smoking status during the first, second, and third trimesters. We included both the use of prenatal care during the first trimester and parity, which is positively associated with the risk of obesity in the literature [6].

#### Statistical analysis

Data on prepregnancy BMI and obesity trends were identified and tested for significance using simple linear regression (SLR) over time. Analysis of variance (AOV) was performed to test the differences in mean birth weight and mean gestational age by six different categories of BMI. Statistical differences among the categories of BMI for both mean birth weight and mean gestational age was tested using an F test followed by an LSD at p = 0.05 (when the F test was significant).

Descriptive statistics were used to characterize the demographic profile of all California resident women with a valid prepregnancy BMI value during the study period. Finally, multivariate logistic regression (MLR) analysis was performed to quantify the association between prepregnancy BMI category and birth outcomes, controlling for probable risk factors including maternal sociodemographic characteristics, health insurance, prenatal care, WIC participation, smoking during pregnancy, and parity. This study investigated the effect of each pregnancy as a unique pregnancy. Births with missing data were handled by exclusion during multivariate analysis.

Calculations were performed for both unadjusted (crude) odds ratios (ORs) and adjusted odds ratios (AORs), with 95% confidence intervals (CIs) and *p*-values and results were reported on AORs. MLR models were used with birth outcome as the outcome variable and maternal prepregnancy BMI category as the exposure variable, controlling for potential covariates. For all models, women with normal BMI were considered to be the reference group and cases with missing data for variables within the model were excluded. The significance level was set at *p* = 0.05. All statistical analyses were performed using SAS software, version 9.3 (SAS Institute Inc., Cary, NC, USA).

## Results

The study population comprised 4,621,082 women with singleton births, selected from 5,054,968 women who had 5,137,376 live births in California from 2007 to 2016. Fig 1 summarizes the screening criteria used to identify the study population (Fig 1). A total of 433,886 women (8.6%) were excluded because of missing, incomplete, or out-of-range data for BMI and records with missing information on gestational age or birth weight.

Almost 49% of the study population (N = 2,260,956) comprised births to women with normal BMI. This was the reference group to which other BMI groups were compared employing MLR. In the study population, 21.1% of the births were to women with obesity, while 26.0% were to overweight women.

The mean age of primiparous women in the study population advanced by 2 years, from 25.6 ± 0.029 years in 2007, to 27.6 ± 0.028 years in 2016. Therefore, birth year was included in the adjustments for the MLR models.

**Fig 2:**
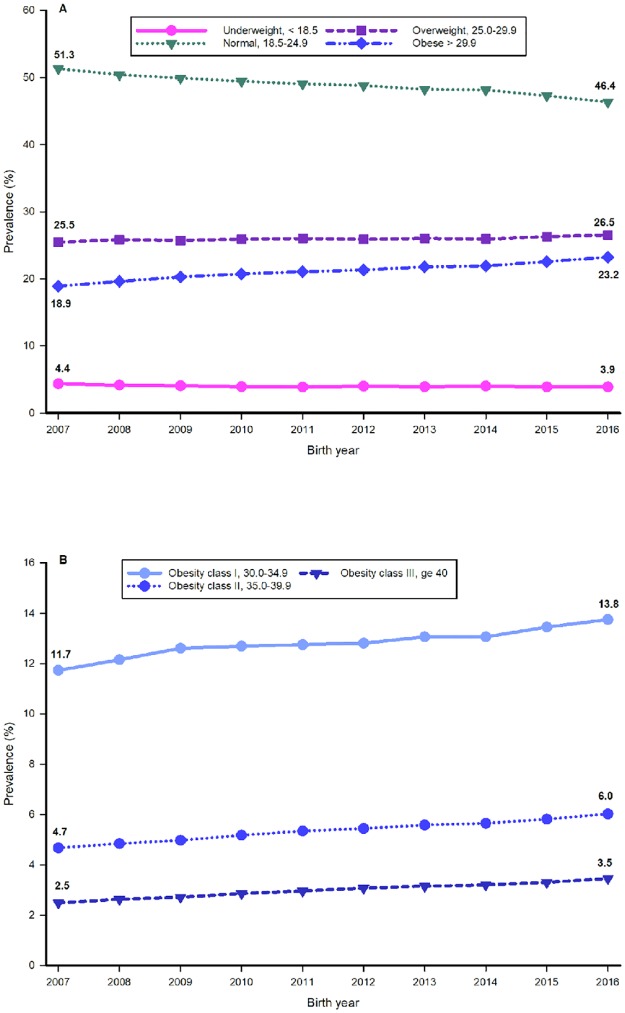
Prevalence (percentage) of body mass index categories in eligible prepregnant women in California from 2007 to 2017. (A) Prevalence for four major prepregnancy body mass index categories: Underweight (< 18.5kg/m^2^), Normal weight (18.5–24.9kg/m^2^), Overweight (25.0–29.9 kg/m^2^), and Obese (≥ 30kg/m^2^) and (B) Prevalence for three subclasses of prepregnancy obesity: Obesity class I (30.0–34.9 kg/m^2^), Obesity class II (35.0–39.9 kg/m^2^), and Obesity class III (≥ 40 kg/m^2^).

Table 1 shows the number and percentage of women in each BMI category for each year from 2007 to 2016. The prevalence of underweight women declined by 10.6% (*p* < .001), from 4.4% in 2007 to 3.9% in 2016. The prevalence of normal weight decreased by 9.7% (*p* < .001), from 51.3% in 2007 to 46.4% in 2016 (Table 1, Fig 2). The prevalence of overweight women increased by 4.3% (*p* < .001), from 25.5% in 2007 to 26.5% in 2016. The prevalence of all obesity classes increased by 22.9% (*p* < .001), from 18.9% in 2007 to 23.2% in 2016 (Table 1, Fig 2, S1 Fig).

**Table 1:** Number and percentage (in parentheses) of eligible^a^ women in California in each prepregnancy body mass index category from 2007 to 2016.

Body Mass Index Category (kg/m^2^)	2007	2008	2009	2010	2011	2012	2013	2014	2015	2016
Underweight, < 18.5	20,606 (4.4)	19,653 (4.2)	18,980 (4.1)	18,104 (3.9)	17,693 (3.9)	18,348 (4.0)	17,700 (3.9)	18,554 (4.0)	17,733 (3.9)	17,686 (3.9)
Normal, 18.5–24.9	243,336 (51.3)	238,798 (50.4)	234,268 (49.9)	228,167 (49.5)	224,067 (49.1)	224,633 (48.8)	218,405 (48.3)	222,539 (48.2)	215,932 (47.3)	210,811 (46.4)
Overweight, 25.0–29.9	120,660 (25.5)	122,227 (25.8)	120,640 (25.7)	119,482 (25.9)	118,841 (26.0)	119,106 (25.9)	117,825 (26.0)	119,786 (25.9)	119,961 (26.3)	120,705 (26.5)
**Obese > 30.0**	**89,557 (18.9)**	**92,881 (19.6)**	**95,199 (20.3)**	**95,539 (20.7)**	**96,188 (21.1)**	**98,075 (21.3)**	**98,587 (21.8)**	**101,261 (21.9)**	**102,955 (22.6)**	**105,594 (23.2)**
Obesity class I, 30.0–34.9	55,596 (11.7)	57,534 (12.2)	59,154 (12.6)	58,520 (12.7)	58,253 (12.8)	58,949 (12.8)	59,102 (13.1)	60,361 (13.1)	61,389 (13.5)	62,536 (13.8)
Obesity class II, 35.0–39.9	22,152 (4.7)	22,904 (4.8)	23,324 (5.0)	23,840 (5.2)	24,396 (5.3)	25,019 (5.4)	25,231 (5.6)	26,093 (5.7)	26,508 (5.8)	27,363 (6.0)
Obesity class III, ≥ 40	11,809 (2.5)	12,443 (2.6)	12,721 (2.7)	13,179 (2.9)	13,539 (3.0)	14,107 (3.1)	14,254 (3.2)	14,807 (3.2)	15,058 (3.3)	15,695 (3.5)

^a^Eligible women in the study population are those who had a live singleton birth delivered at 17–47 weeks of gestation based on obstetric estimates and birthweight of ≥ 500 g at birth and with valid information for BMI. This defines the study population consisting of 4,621,082 women.

Obesity class I increased by 17.2% (*p* < .001), from 11.7% in 2007 to 13.8 in 2016, while obesity class II increased by 28.9% (*p* < .001), from 4.7% in 2007 to 6.0 in 2016. The greatest increase was seen in obesity class III, which increased by 38.6% (*p* < .001), from 2.5% in 2007 to 3.5 in 2016 (Table 1, Fig 2). The entire maternal population progressed toward a higher BMI (Table 1, S1 Fig). The numbers and percentages of women in all BMI categories according to the covariates described in this study for the period 2007 to 2016 are shown in Table 2. Of the women who were overweight or obese before pregnancy, more than 50% were 25 to 34 years of age. Of the obese women, over 62% were Hispanic and almost 84% did not attain an education equivalent to a bachelor’s degree or higher. In this cohort, more women who were born in the United States were obese than women born elsewhere. Almost 60% of women who received Medi-Cal insurance as the funding source for their prenatal care were obese before pregnancy. Of the WIC recipients, more than 64% were obese before becoming pregnant (Table 2). Smoking prevalence rose with advancing obesity classes (Table 2).

**Table 2:** Number and percentage (in parentheses) for each category of maternal characteristic and health behavior for each category of body mass index for eligible* prepregnant women in California for the period 2007–2016.

Variables	Body Mass Index categories (kg/m^2^)						
	Underweight, <18.5	Normal, 18.5–24.9	Overweight, 25.0–29.9	Obese, ≥ 30.0	Obesity classes		
					Obesity class I, 30.0–34.9	Obesity class II, 35.0–39.9	Obesity class III, ≥40
***Maternal age*, *years***							
<20	23,448(12.7)	193,871(8.6)	78,121(6.5)	45,684 (4.7)	31,421(5.3)	10,209(4.1)	4,054(3.0)
20–24	44,616(24.1)	443,861(19.6)	246,445(20.6)	206,504 (21.2)	126,000(21.3)	52,609(21.3)	27,895(20.3)
25–29	47,196(25.5)	574,148(25.4)	329,755(27.5)	285,092 (29.2)	168,399(28.5)	73,563(29.8)	43,130(31.3)
30–34	43,825(23.7)	619,173(27.4)	316,523(26.4)	256,494 (26.3)	153,329(25.9)	65,440(26.5)	37,725(27.4)
35–39	21,321(11.5)	344,600(15.2)	179,605(15.0)	143,675 (14.7)	87,920(14.9)	35,698(14.5)	20,057(14.6)
40–54	4,650(2.5)	85,199(3.8)	48,689(4.1)	38,346 (3.9)	24,295(4.1)	9,302(3.8)	4,749(3.5)
***Maternal race/ethnicity***							
Hispanic	59,761(32.3)	933,560(41.3)	691,468(57.7)	608,026 (62.3)	373,552(63.2)	152,534(61.8)	81,940(59.5)
White^†^	52,381(28.3)	718,418(31.8)	284,712(23.7)	211,717 (21.7)	123,912(21.0)	55,998(22.7)	31,807(23.1)
Asian^‡^	55,129(29.8)	404,495(17.9)	104,810(8.7)	37,964 (3.9)	29,224(4.9)	6,794(2.8)	1,946(1.4)
Pacific Islander^§^	357(0.2)	5,227(0.2)	5,174(0.4)	8,475 (0.9)	4,203(0.7)	2,580(1.1)	1,692(1.2)
African American	9,075(4.9)	94,307(4.2)	62,648(5.2)	67,239 (6.9)	35,888(6.1)	17,991(7.3)	13,360(9.7)
Multiple race	4,135(2.2)	49,051(2.2)	23,648(2.0)	21,324 (2.2)	12,012(2.0)	5,645(2.3)	3,667(2.7)
American Indian^||^	452(0.2)	5,855(0.3)	4,191(0.4)	5,426 (0.6)	2,813(0.5)	1,490(0.6)	1,123(0.8)
Other/unknown^¶^	3,767(2.0)	50,043(2.2)	22,582(1.9)	15,665 (1.6)	9,790(1.7)	3,798(1.5)	2,077(1.5)
***Maternal educational level***							
Less than high school	31,064(16.8)	381,440(16.9)	286,061(23.9)	233,251 (23.9)	149,820(25.3)	55,396(22.4)	28,035(20.4)
High school diploma	45,139(24.4)	504,113(22.3)	316,735(26.4)	295,732 (30.3)	173,038(29.3)	77,075(31.2)	45,619(33.2)
Some college/associate degree	41,293(22.3)	510,333(22.6)	299,140(24.9)	282,426 (28.9)	161,558(27.3)	75,364(30.5)	45,504(33.1)
Bachelor's degree or higher	60,110(32.5)	774,895(34.3)	252,528(21.1)	132,112 (13.5)	86,606(14.6)	31,144(12.6)	14,362(10.4)
Unknown	7,451(4.0)	90,175(4.0)	44,769(3.7)	32,315 (3.3)	20,372(3.4)	7,851(3.2)	4,092(3.0)
***Maternal nativity***							
Foreign-born	87,682(47.4)	941,104(41.6)	503,916(42.0)	320,737 (32.9)	221,198(37.4)	70,496(28.6)	29,043(21.1)
US-born	97,305(52.6)	1,319,033(58.4)	695,070(58.0)	654,983 (67.1)	370,128(62.6)	176,310(71.4)	108,545(78.9)
***Maternal demographic region***							
Central Coast	8,012(4.3)	128,390(5.7)	80,111(6.7)	61,222 (6.3)	38,453(6.5)	15,101(6.1)	7,668(5.6)
Greater Bay Area	34,039(18.4)	443,733(19.6)	196,942(16.4)	139,255 (14.3)	85,587(14.5)	34,509(14.0)	19,159(13.9)
Inland Empire	22,389(12.1)	255,164(11.3)	154,816(12.9)	141,473 (14.5)	83,147(14.1)	36,841(14.9)	21,485(15.6)
Los Angeles County	51,887(28.0)	575,720(25.5)	302,707(25.2)	242,529 (24.9)	150,093(25.4)	60,191(24.4)	32,245(23.4)
Northern and Sierra	5,534(3.0)	67,896(3.0)	36,850(3.1)	35,042 (3.6)	19,724(3.3)	9,369(3.8)	5,949(4.3)
Orange County	18,729(10.1)	195,029(8.6)	84,431(7.0)	57,894 (5.9)	36,879(6.2)	14,086(5.7)	6,929(5.0)
Sacramento Area	8,966(4.8)	120,165(5.3)	63,497(5.3)	54,434 (5.6)	31,585(5.3)	14,261(5.8)	8,588(6.2)
San Diego Area	17,093(9.2)	237,381(10.5)	114,930(9.6)	83,490 (8.6)	52,075(8.8)	20,653(8.4)	10,762(7.8)
San Joaquin Valley	18,408(10.0)	237,478(10.5)	164,949(13.8)	160,497 (16.5)	93,851(15.9)	41,819(16.9)	24,827(18.0)
***Source of prenatal care payment***							
Private insurance	86,661(52.6)	1,187,942(57.4)	521,184(46.7)	389,546 (42.4)	235,006(42.3)	100,022(42.9)	54,518(41.8)
Medi-Cal (Public)	78,143(47.4)	883,137(42.6)	595,388(53.3)	529,937 (57.6)	320,981(57.7)	133,103(57.1)	75,853(58.2)
***Supplemental Nutrition Program for Women*, *Infants*, *and Children (WIC) food recipients***							
No	100,395(54.3)	1,266,864(56.0)	514,232(42.9)	350,147 (35.9)	216,949(36.7)	87,618(35.5)	45,580(33.1)
Yes	84,662(45.8)	994,092(44.0)	685,001(57.1)	625,689 (64.1)	374,445(63.3)	159,212(64.5)	92,032(66.9)
***First trimester prenatal care initiation***							
No	32,829(18.0)	350,744(15.7)	204,752(17.3)	174,877 (18.1)	105,607(18.1)	44,158(18.1)	25,112(18.5)
Yes	149,175(82.0)	1,880,227(84.3)	980,176(82.7)	789,077 (81.9)	478,678(81.9)	199,678(81.9)	110,721(81.5)
***Parity***							
Primiparous	100,045(54.1)	1,026,921(45.5)	415,363(34.7)	289,266 (29.7)	177,855(30.1)	71,985(29.2)	39,426(28.7)
Multiparous (2–5)	83,464(45.1)	1,206,323(53.4)	755,094(63.0)	652,926 (67.0)	394,068(66.7)	165,992(67.3)	92,866(67.6)
Multiparous (6–12)	1,392(0.8)	25,925(1.2)	27,710(2.3)	32,664 (3.4)	18,874(3.2)	8,623(3.5)	5,167(3.8)
***Maternal smoking during both first and second trimesters***							
No	177,965(97.5)	2,206,233(98.6)	1,167,568(98.6)	944,005 (98.2)	573,430(98.4)	238,370(98.1)	132,205(97.8)
Yes	4,611(2.5)	31,753(1.4)	17,058(1.4)	17,252 (1.8)	9,515(1.6)	4,690(1.9)	3,047(2.3)

*Eligible women are those who had a live Singleton birth delivered at 17–47 weeks of gestation based on obstetric estimates and birthweight of ≥ 500 g at birth and with valid information for BMI. This defines the study population consisting of 4,621,082 women

Race-ethnicity results were tabulated using the following racial and ethnic groups: Hispanic, White, Asian/ Pacific Islander, African American, Multiple race (2 or more races), American Indian, and other

Hispanic origin was determined first and could include any racial group. Women who were members of two or more racial groups were not reported in the single-race groups. To remain consistent with the population data obtained from the California Department of Finance, the single-race groups are defined as follows:

^†^ “White” race: white women

^‡^ “Asian” race: Asian Indian, Asian (specified or unspecified), Cambodian, Chinese, Filipino, Hmong, Japanese, Korean, Laotian, Thai, and Vietnamese women

^§^ “Pacific Islander” race: Guamanian, Hawaiian, Samoan, and other Pacific Islander women

^||^ “American Indian” race: Aleutian, American Indian, and Eskimo women

^¶^ “Other/unknown” race: race not stated or unknown

**Fig 3:**
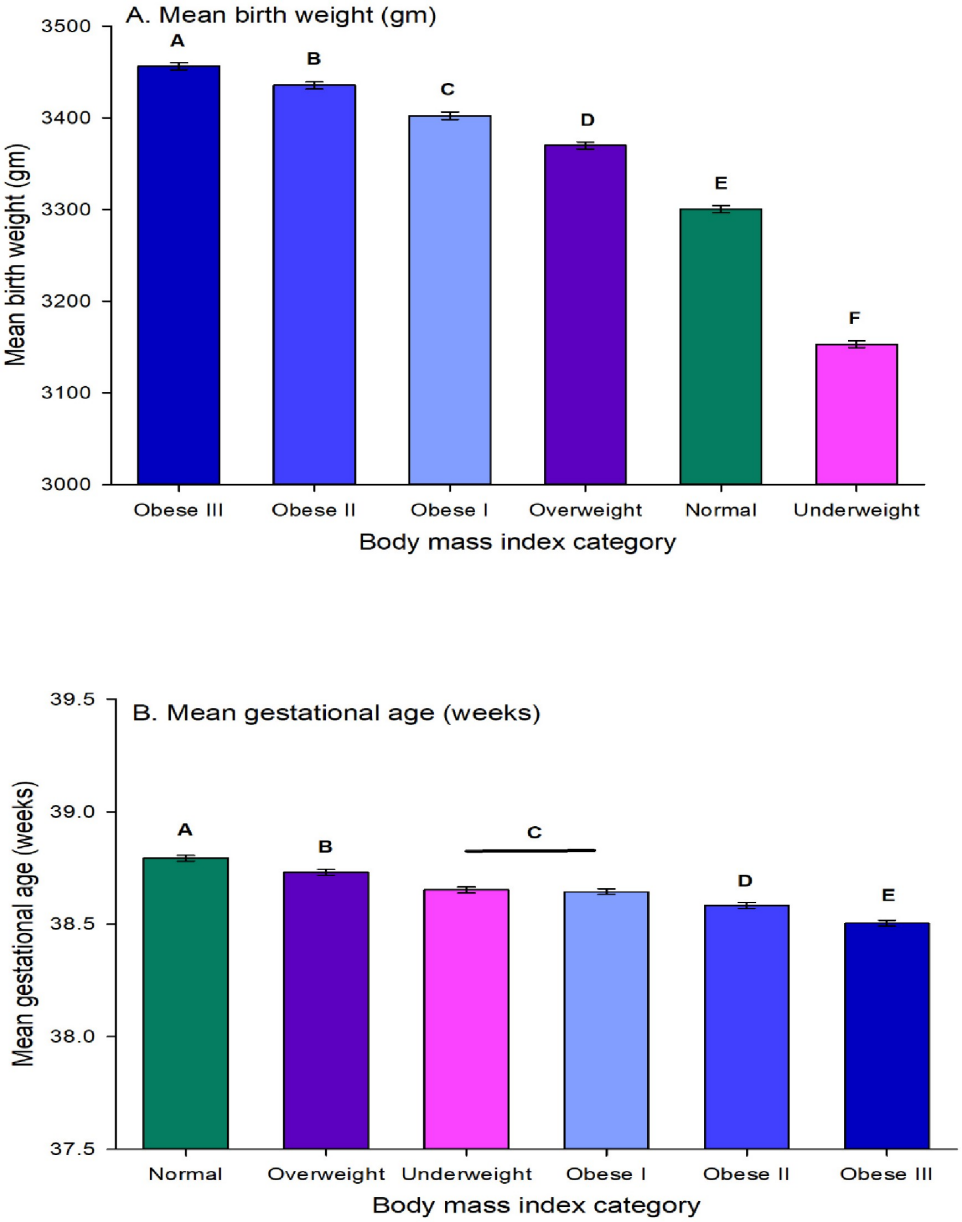
Mean birth weight in grams (A) and mean gestational age in weeks (B) by six prepregnancy obesity groups from 2007 to 2016 (with error bars indicating minimum significant difference for Tukey's studentized range at p = 0.05).

Infants born to underweight women had lighter birth weight while obese women had heavier birth weight infants. Mean birth weight significantly (*p* = .007) increased from women who were underweight (3,153.2 gm) to women with obesity class III (3,456.4 gm) and each class was significantly different from the other classes (*p* < .001) (Fig 3A).

Contrary to mean birth weight, mean gestational age significantly decreased (*p* < .001) from women with normal weight (38.8 weeks) to women with obesity class III (38.5 weeks) and each class was significantly different from the other classes (*p* < .001) (Fig 3B), except for underweight and obese class I women.

Table 3 describes the numbers and percentages of three categories of intrauterine growth stage, SGA, AGA, and LGA for all births at 23–41 weeks of gestation based on obstetric estimates (OEs).

**Table 3:** Number of all births at 23–41 weeks of gestation based on obstetric estimates (OEs) for the three categories of intrauterine growth stage for the 4,594,570 such births in California from 2007 to 2016, with the row wise percentage of such infants in parenthesis.

BMI category	SGA	AGA	LGA
**Underweight, <18.5**	17,738 (9.6)	161,820 (87.9)	4,621 (2.5)
**Normal, 18.5–24.9**	131,843 (5.9)	2,003,161 (89.1)	112,529 (5.0)
**Overweight, 25.0–29.9**	55,724 (4.7)	1,039,682 (87.2)	96,989 (8.1)
**Obese I,II,III ≥ 30.0**	**40,451 (4.2)**	**813,343 (83.8)**	**116,669 (12.0)**
**Obese I, 30.0–34.9**	25,365 (4.3)	499,934 (85.0)	62,889 (10.7)
**Obese II, 35.0–39.9**	9,825 (4.0)	203,183 (82.8)	32,477 (13.2)
**Obese III, ≥ 40**	5,261 (3.9)	110,226 (80.6)	21,303 (15.6)
**Total**	**245,756 (5.4)**	**4,018,006 (87.5)**	**330,808 (7.2)**

Preterm: < 37 weeks of gestation; Term: ≥37 weeks of gestation; *SGA* small-for-gestational-age, *AGA* appropriate-for-gestational-age, *LGA* large-for-gestational-age, *LBW* Low birth weight (< 2500 g)

### Birth outcomes

**Fig 4:**
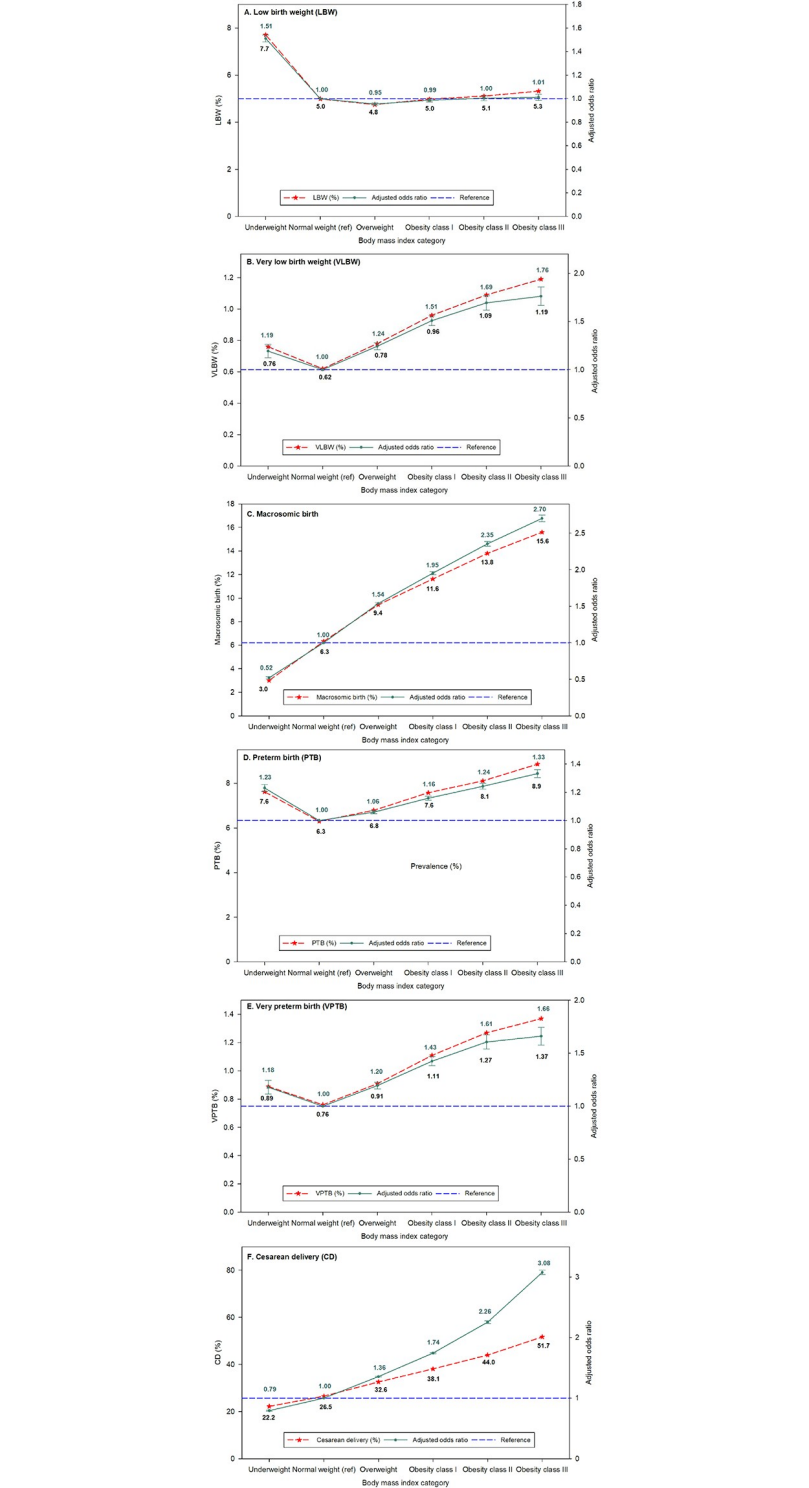
Prevalence (percentage) and adjusted odds ratio for 6 outcomes associated with body mass index in eligible prepregnant women in California for the period 2007–2016. A. Low birth weight, B. Very low birth weight, C. Macrosomic birth, D. Preterm birth, E. Very preterm birth, and F. Cesarean delivery.

**Fig 5:**
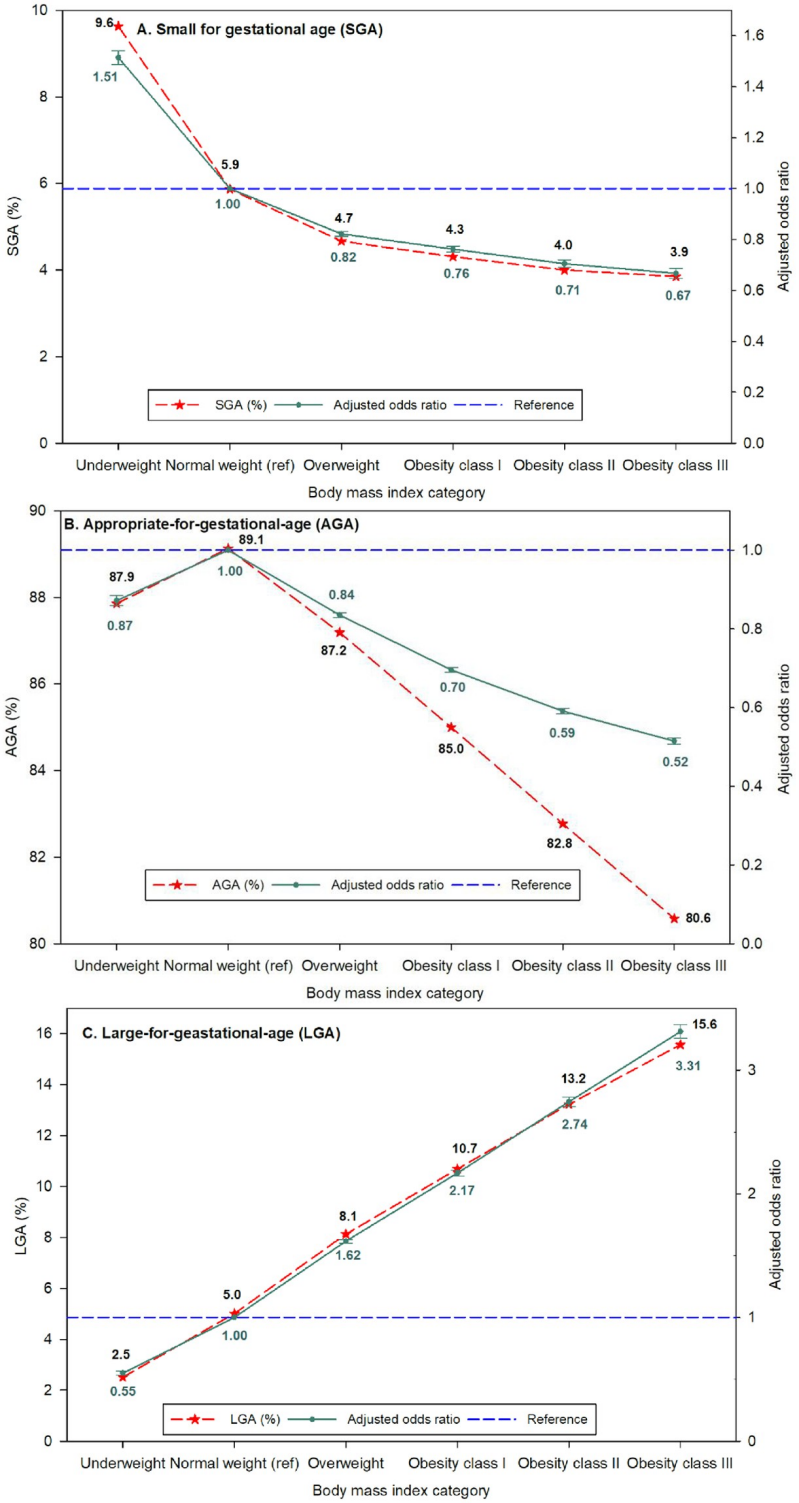
Percentage and adjusted odds ratio for IUGR outcomes of infants associated with body mass index in eligible prepregnant women in California for the period 2007–2016. A. Small-for-gestational-age (SGA) B. Appropriate-for-gestational-age (AGA), and C. Large-for-gestational-age (LGA).

The results of MLR analysis models, with birth outcomes as the outcome variables and the six maternal BMI categories as the exposure variables controlling for study covariates, are given in Table 4, Figs 4 and 5).

**Table 4:** Multivariate-adjusted independent effects of maternal prepregnancy body mass index category on birth and maternal outcomes for eligible women in California for the period 2007–2016.

Birth–or maternal outcome	Body Mass Index category (kg/m^2^)										
	Underweight (< 18.5)		Normal weight (18.5–24.9)	Overweight (25.0–29.9)		Obesity class I (30.0–34.9)		Obesity class II (35.0–39.9)		Obesity class III (≥ 40)	
**LBW**	**1.51 (1.48–1.54)**	**< .001**	ref	**0.95 (0.94–0.97)**	**< .001**	0.99 (0.97–1.00)	0.071	1.00 (0.98–1.02)	0.721	1.01 (0.99–1.04)	0.373
**VLBW**	**1.19 (1.12–1.27)**	**< .001**	ref	**1.24 (1.21–1.28)**	**< .001**	**1.51 (1.46–1.56)**	**< .001**	**1.69 (1.62–1.77)**	**< .001**	1.76 (1.67–1.86)	**< .001**
**Macrosomic birth**	**0.52 (0.51–0.54)**	**< .001**	**ref**	**1.54 (1.53–1.55)**	**< .001**	**1.95 (1.93–1.97)**	**< .001**	**2.35 (2.32–2.39)**	**< .001**	**2.70 (2.66–2.75)**	**< .001**
**PTB**	**1.23 (1.21–1.26)**	**< .001**	**ref**	**1.06 (1.05–1.07)**	**< .001**	**1.16 (1.14–1.17)**	**< .001**	**1.24 (1.22–1.26)**	**< .001**	**1.33 (1.30–1.36)**	**< .001**
**VPTB**	**1.18 (1.12–1.25)**	**< .001**	**ref**	**1.20 (1.16–1.23)**	**< .001**	**1.43 (1.38–1.47)**	**< .001**	**1.61 (1.54–1.68)**	**< .001**	**1.66 (1.58–1.75)**	**< .001**
**SGA**	**1.51 (1.49–1.54)**	**< .001**	**ref**	**0.82 (0.81–0.83)**	**< .001**	**0.76 (0.75–0.77)**	**< .001**	**0.71 (0.69–0.72)**	**< .001**	**0.67(0.65–0.69)**	**< .001**
**AGA**	**0.87 (0.86–0.89)**	**< .001**	**ref**	**0.84 (0.83–0.84)**	**< .001**	**0.70 (0.69–0.70)**	**< .001**	**0.59 (0.58–0.60)**	**< .001**	**0.52 (0.51–0.52)**	**< .001**
**LGA**	**0.55 (0.53–0.57)**	**< .001**	**ref**	**1.62 (1.60–1.63)**	**< .001**	**2.17 (2.14–2.19)**	**< .001**	**2.74 (2.71–2.78)**	**< .001**	**3.31 (3.26–3.37)**	**< .001**
**Cesarean delivery**	**0.79 (0.78–0.80)**	**< .001**	**ref**	**1.36 (1.35–1.36)**	**< .001**	**1.74 (1.73–1.76)**	**< .001**	**2.26 (2.24–2.28)**	**< .001**	**3.08 (3.04–3.11)**	**< .001**

Results in bold indicate statistical significance (*p* < .05)

Data are expressed as adjusted odds ratio (95% confidence interval) with *p* value (using the chi-square test). Multivariate logistic regression models controlled for maternal age, race and ethnicity, education level, nativity, demographic region, source of prenatal care payment, Federal Supplemental Nutrition Program for Women, Infants, and Children participation, first-trimester prenatal care initiation, parity, and maternal smoking status

LBW: low birth weight; VLBW: very low birth weight; PTB: preterm birth; VPTB: very preterm birth; SGA: small-for-gestational-age; AGA: appropriate-for-gestational-age, and LGA: large-for-gestational-age

#### LBW and VLBW

The unadjusted incidence of LBW in underweight women was 7.7% (Fig 4A). Underweight women were 51% more likely (95% CI = 1.48–1.54) to have an LBW infant compared with normal-weight women (Table 4, Fig 4A). We found no significant association between incidence of LBW and BMI categories excluding underweight category (Table 4).

There was a highly significant association (*p* < .001) between incidence of VLBW and BMI category. Using women of normal weight as the reference, the incidence of VLBW increased with increasing BMI, by 24% in overweight women (95% CI = 1.21–1.28) to 76% in women with class III obesity (95% CI = 1.67–1.86) (Fig 4B). Women who were underweight were 19% more likely (95% CI = 1.12–1.27) to have a VLBW infant than were women of normal weight. The unadjusted incidence also showed the same pattern, with the highest incidence of 1.19% in obese class III women.

#### Macrosomic birth

In our study population, 8.3% of the infants were macrosomic. The incidence of macrosomic birth was lowest in underweight women (3.0%) and increased linearly with increasing maternal prepregnancy BMI category; the highest percentage, 15.6%, was in women with class III obesity (Fig 4C). Compared with women of normal weight, women with class I obesity had almost double the likelihood of having a macrosomic birth (AOR = 1.95; 95% CI = 1.93–1.97). The likelihood of having a macrosomic birth was even higher in women with class II obesity (AOR = 2.35; 95% CI = 2.32–2.39) and class III obesity (AOR = 2.70; 95% CI = 2.66–2.75) (Table 4, Fig 4C).

#### PTB and VPTB

The incidence of PTB and VPTB rose with increasing BMI category above normal weight, with the highest rate of PTB, 8.9%, and the highest rate of VPTB, 1.37%, in women with class III obesity (Fig 4D and 4E). Multivariate logistic regression analysis, using women of normal weight as the reference, found significant associations between incidence of PTB and BMI category, and incidence of VPTB and BMI category (Table 2, Fig 4D and 4E). Women who were underweight were 23% more likely (95% CI = 1.21–1.26) to have a PTB infant and 18% more likely (95% CI = 1.12–1.25) to have a VPTB infant than were women of normal weight (Table 4, Fig 3D and 3E). Women who were obese class I were 16% more likely (95% CI = 1.14–1.17) to have a PTB and 43% more likely (95% CI = 1.38–1.47) to have a VPTB infant than were women of normal weight (Table 4). Furthermore, women with obesity class III were 33% more likely (95% CI = 1.30–1.36) to have a PTB and 66% more likely (95% CI = 1.58–1.75) to have a VPTB infant than were women with a normal BMI (Table 4, Fig 4D and 4E).

#### SGA, AGA, and LGA

The SGA, AGA, and LGA infants comprised 5.4%, 87.5%, and 7.2%, respectively in the study population (Table 3). Among macrosomic infants, 66.7% of the infants were LGA.

After adjusting for potential confounders, births to underweight women were 51% (95% CI = 1.49–1.54) more likely to have SGA infants and 12.8% (95% CI = 0.86–0.89 less likely to have AGA infants than women with normal weight (Table 4, Fig 5A).

AGA infants were less likely among women of all maternal prepregnancy categories when compared with women of normal prepregnancy weight (Table 4, Fig 5B).

Moreover, LGA infants were more likely among women who were overweight 62% (95% CI = 1.60–1.63), obesity class I 117% (95% CI = 2.14–2.19), obesity class II 174% (95% CI = 2.71–2.78), and obesity class III 231% (95% CI = 3.26–3.37) than among women with normal weight, respectively (Table 4, Fig 5C).

### Cesarean delivery

The rate of cesarean delivery was found to increase with increasing maternal BMI, from 22.2% in women who were underweight to 51.7% in women in obesity class III (Fig 4F).

The incidence of cesarean delivery was significantly associated with BMI category, with the lowest rate seen in underweight women. It was 36% higher (95% CI = 1.35–1.36) in overweight mothers than in those of normal weight (Table 2, Fig 4F), and more than 3-fold higher (AOR = 3.08; 95% CI = 3.04–3.11) in women with class III obesity (Table 2, Fig 4F). The outcomes from analyzing first, second and third births were consistent with analyzing each pregnancy (S1–S3 Tables).

## Discussion

Our study shows that, from 2007 to 2016, the trends in underweight and normal weight declined as women drifted toward the higher BMI categories. As a result, the prevalence of the 3 classes of prepregnancy obesity increased in an almost linear fashion. The Healthy People 2020 objectives include increasing the proportion of women who become pregnant at a normal weight, from 52.5% in 2007 to 57.8% by 2020 [5]. However, Deputy et al. observed that the prevalence of normal weight has decreased by 5%, whereas the prevalence of overweight has increased by 2% and the prevalence of all classes of obesity has increased by 8% from 47.3% to 45.0%, from 2011 to 2015 in 38 jurisdictions in United States [5]. These results suggest that the Healthy People 2020 target for prepregnancy normal weight will not be attained, as trends are going in the wrong direction [5]. The findings of the present study show an increasing prevalence of maternal prepregnancy women who are overweight and in all classes of obesity. Our findings are consistent with those of Branum and colleagues, who analyzed data from 47 states and the District of Columbia in the U.S. Standard Certificate of Live Birth for 2014 [8].

Previously published studies have documented the increased risk of adverse birth outcomes associated with maternal obesity during pregnancy [22,29,30]. However, few studies have provided quantitative estimates for the association of poor birth outcomes with the category of maternal prepregnancy BMI using a large dataset such as the BSMF. We found no significant difference in the incidence of LBW among prepregnancy women who were overweight and those in the three obesity classes [31]. However, increasing maternal prepregnancy BMI is strongly associated with an increased risk of VLBW [31].

Fetal macrosomia is another complication seen in women with prepregnancy obesity [32,33]. Our findings support those of Moussa et al from 2016 [10], in that the rate of macrosomic births increased linearly with increasing maternal prepregnancy BMI category. The presence of macrosomia contributes to adverse outcomes for both mother and child, including an increased risk of complications during labor, increased birth injuries associated with delivery interventions, and increased neonatal morbidity and mortality [34–37].

We know that PTB is the leading cause of infant mortality, neonatal morbidity, and long-term disability; these risks increase with decreasing gestational age [38,39]. Both low and high BMI have been shown to be associated with PTB [40,41]. Girsen et al. found that an underweight maternal prepregnancy BMI was associated with an increased risk-adjusted rate of PTB [42]. A data synthesis conducted by McDonald et al in 2010, based on 84 studies and totaling 1 095 834 women, found that overweight and obese women have an increased risk of PTB and induced PTB, after accounting for publication bias [42]. Obesity increases the risk of medically indicated preterm delivery [42–44]. Several previously published studies have linked this causation to obesity-related maternal complications, including pre-eclampsia [29,45,46]. Our findings are consistent with studies conducted in Sweden, which also find that maternal overweight and obesity during pregnancy is associated with an increased risk for PTB, especially VPTB [23]. In addition to the increased risk of PTB and VPTB conferred by abnormal BMI, it has also been shown that for those infants born preterm, abnormal BMI confers a higher risk of morbidities associated with PTB compared to preterm infants born to women with normal BMI [47,48].

We observed significant differences in fetal growth among the six different BMI categories of prepregnant women. In this study, underweight women were more likely to have SGA infants and their mean birthweight was lower than other BMI categories. Early age at pregnancy and short interpregnancy intervals were also associated with increased risk of PTB, LBW, SGA, and neonatal death [49,50]. Maternal undernutrition contributes to neonatal deaths through SGA [51] and short and long term health outcomes [52].

We were unable to control for interpregnancy intervals to quantify confounders for birth outcomes evaluated, which is a study limitation. Poor nutritional status in fetus is harmful to the development and function of the unborn infant, predisposing them to the development of adult chronic diseases which is popularly known as the Barker hypothesis [53,54]. Maternal nutrition, both before and during pregnancy is likely to play a crucial role to improve pregnancy outcomes [55] and has important implications on subsequent maternal and offspring health, including outcomes in later adult life [56].

Our study also showed that the incidence of LGA infants increases with increasing BMI level from overweight to obesity class III. Recent animal studies revealed that excessive nutrition also increased the risk of cardiovascular diseases among offspring [54]. Either insufficient or excessive nutrition alters epigenetic modification of genes that encodes enzymes associated with lipid metabolism [54]. This altered epigenetic state persists during one’s lifetime and may potentially lead to noncommunicable adulthood diseases [54].

Several previously published studies have reported that women who are overweight or obese have an increased risk of cesarean delivery compared with women of normal weight [11]. Ours is the first study to examine the strength and extent of this relationship in a modern California cohort. We found a strong association between cesarean delivery and BMI category, using normal weight as the reference. The incidence of cesarean delivery increases progressively with increasing maternal BMI, from overweight to class III obesity. The AORs in our study are similar to those reported in a meta-analysis of 33 studies on maternal obesity and risk of cesarean delivery reported by Chu et al in 2007 [11], but with smaller confidence intervals because of our larger study cohort. Chu et al found that the unadjusted ORs of a cesarean delivery are 1.46 (95% CI = 1.34–1.60), 2.05 (95% CI = 1.86–2.27), and 2.89 (95% CI = 2.28–3.79) in overweight, obese, and severely obese women, respectively, compared with normal-weight pregnant women [11].

Dude et al, [57], who showed that weight management in the postpartum period, during the interval between pregnancies, and during subsequent pregnancy helps to reduce the risk of future cesarean delivery. They also found that infants delivered by cesarean have a higher BMI at 6 months of age than infants who were born vaginally, suggesting that obesity affects both mothers and their offspring [57].

Our study has several strengths, including a large sample size of more than 4.6 million women who delivered singleton live births over the most recent 10-year period, from a highly diverse population in California. Our data include variations in geography, healthcare funding as a reflection of socioeconomic status, and smoking status during pregnancy as covariates, which previous studies were unable to analyze. These advantages represent those of a large, statewide study conducted over a 10-year period in California.

Our study has several limitations. Prepregnancy weight is self-reported or abstracted from medical records, which might lead to incorrect classification in BMI categories. However, previous work based on the National Health and Nutrition Examination Survey agree with information being derived from birth certificate data [7,58,59].

Another limitation is maternal sociodemographic characteristics are self-reported or abstracted from birth certificates and may contain inaccuracies. However, Dietz, et al (2014) proved the reliability of maternal demographic information and gestational age at birth recorded in the birth certificate [60].

Finally, some confounding variables may not exist in the California BSMF. We were unable to include paternal/partner influence and other socioeconomic inequalities (e.g.: housing, job security etc.) in maternal prepregnancy obesity. Risk profiles are not straightforward and often involve issues of lifestyle, adherence to medical advice, disparities in social determinants of health, and other behaviors that are hard to modify, as described by Schroeder [61].

A statistical limitation of our analysis is that we were not able to link pregnancies across the study period to individual women. Some women have been counted more than once in the analysis. This study was based on unique pregnancies, not unique women. We minimized this limitation by controlling for birth year. However, we theorized that each pregnancy is unique and there may be value in investigating this further with appropriate datasets.

From 2007 to 2016 in California, prepregnant women showed a declining trend in normal weight but a rising trend in overweight and obesity categories, particularly in obesity class III. Increasing prepregnancy BMI is associated with increased incidence of VLBW, macrosomia, PTB, VPTB, and cesarean delivery. The recent trend of increasing prepregnancy maternal BMI is a serious public health concern for both mothers and infants. As Stewart et al pointed out in 2009, if we do not take action to stop the rising prevalence of obesity, the negative effects on the health of the US population will increasingly outweigh the positive effects gained from declining smoking rates [62].

They also note that failure to prevent the increasing prevalence of obesity could reverse any improvements in public health, including child health, which have been steadily accruing since the beginning of the twentieth century [62]. The steadily rising rate of prepregnancy obesity in women of childbearing age is a major public health concern, particularly for women with class III obesity. It is important that public health policymakers recognize the rising level of obesity and its effects on birth outcomes, the health of both infants and mothers, and healthcare-associated costs.

Providing the most recent trends for maternal BMI and noting the effects of maternal BMI on birth outcomes using the most recent large datasets will provide crucial information for decision-making by policymakers. Considering ever emerging support and evidence for the Barker hypothesis, pregnancy is the best opportunity to address future health. Therefore, investment in nutrition-specific interventions to prevent both maternal undernutrition and nutrition leading to obesity during pregnancy may help avoid the costly complex health and social needs arising from poor birth outcomes. The time has come to realize that healthy pregnancy is a foundation for the health of the future generation and a key solution to ever-rising health care costs.

## Supporting information

S1 Fig Percentage change in prevalence for each prepregnancy body mass index category for eligible prepregnant women in California from 2007 to 2016. (DOCX)

S1 Table Multivariate-adjusted independent effects of maternal prepregnancy body mass index category on birth and maternal outcomes for the first birth for eligible (primiparous) women in California for the period 2007–2016. (DOCX)

S2 Table Multivariate-adjusted independent effects of maternal prepregnancy body mass index category on birth and maternal outcomes for the second birth for eligible women in California for the period 2007–2016. (DOCX)

S3 Table Multivariate-adjusted independent effects of maternal prepregnancy body mass index category on birth and maternal outcomes for the third birth for eligible women in California for the period 2007–2016. (DOCX)

S1 Checklist STROBE statement for observational studies. (DOCX)
