# Supplementary material for: Trends in maternal prepregnancy body mass index (BMI) and its association with birth and maternal outcomes in California, 2007–2016: A retrospective cohort study
Source: PLoS One. 2019 Sep 19;14(9):e0222458. doi: 10.1371/journal.pone.0222458 (PMC6752764; doi:10.1371/journal.pone.0222458)
Supplement: S1 Fig — (DOCX) [file pone.0222458.s001.docx]

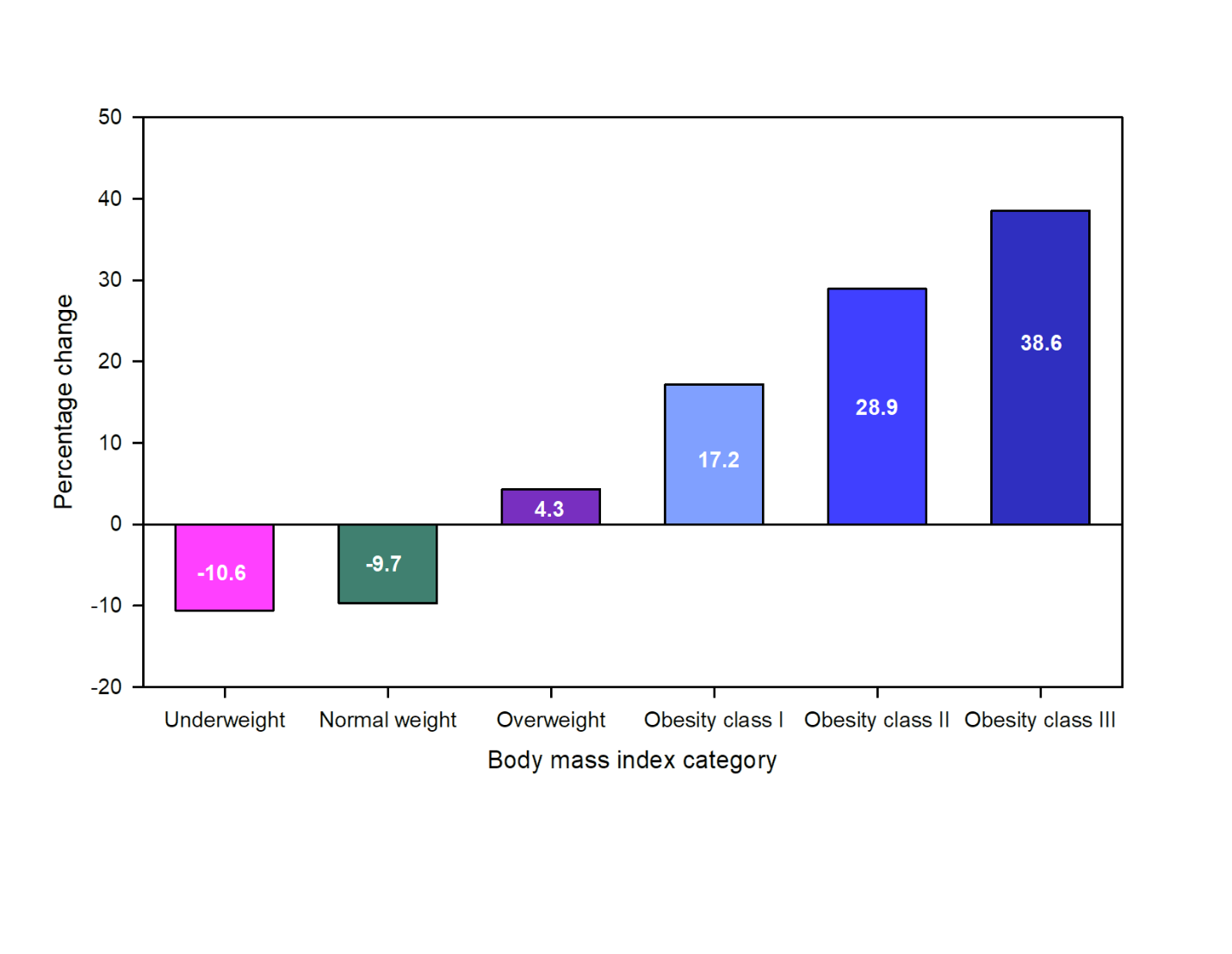


**S1 Fig.** Percentage change in prevalence for each prepregnancy body mass index category for eligible prepregnant women in California from 2007 to 2016
