# Supplementary material for: Trends in maternal prepregnancy body mass index (BMI) and its association with birth and maternal outcomes in California, 2007–2016: A retrospective cohort study
Source: PLoS One. 2019 Sep 19;14(9):e0222458. doi: 10.1371/journal.pone.0222458 (PMC6752764; doi:10.1371/journal.pone.0222458)
Supplement: S3 Table — (DOCX) [file pone.0222458.s004.docx]

**S3 Table.** Multivariate-adjusted independent effects of maternal prepregnancy body mass index category on birth and maternal outcomes for the third birth for eligible women in California for the period 2007–2016.

| **Body Mass Index category (kg/m^2^)** | **Birth- or maternal outcome** | | | | | |
| --- | --- | --- | --- | --- | --- | --- |
|  | **LBW** | **VLBW** | **Macrosomic birth** | **PTB** | **VPTB** | **Cesarean delivery** |
| Underweight (< 18.5) | **1.68 (1.58–1.79)** | **1.26 (1.04–1.52)** | **1.37 (1.30–1.45)** | **1.28 (1.08–1.52)** | **0.81 (0.78–0.84)** | **0.50 (0.46–0.54)** |
|  | **<.001** | **0.016** | **<.001** | **0.004** | **<.001** | **<.001** |
| Normal weight (18.5-24.9) | ref | ref | ref | ref | ref | ref |
|  |  |  |  |  |  |  |
| Overweight (25.0-29.9) | **0.92 (0.89–0.95)** | **1.10 (1.02–1.19)** | **1.03 (1.00–1.05)** | 1.06 (0.99–1.14) | **1.31 (1.29–1.33)** | **1.54 (1.51–1.58)** |
|  | **<.001** | **0.010** | **0.024** | 0.075 | **<.001** | **<.001** |
| Obesity class I (30.0-34.9) | **0.92 (0.89–0.95)** | **1.32 (1.21–1.44)** | **1.08 (1.05–1.11)** | **1.24 (1.14–1.34)** | **1.69 (1.67–1.72)** | **1.96 (1.92–2.01)** |
|  | **<.001** | **<.001** | **<.001** | **<.001** | **<.001** | **<.001** |
| Obesity class II (35.0-39.9) | **0.93 (0.89–0.98)** | **1.40 (1.25–1.57)** | **1.15 (1.11–1.19)** | **1.45 (1.31–1.60)** | **2.15 (2.11–2.20)** | **2.42 (2.35–2.49)** |
|  | **0.005** | **<.001** | **<.001** | **<.001** | **<.001** | **<.001** |
| Obesity class III (≥ 40) | **0.91 (0.86–0.97)** | **1.42 (1.23–1.64)** | **1.28 (1.22–1.34)** | **1.30 (1.14–1.48)** | **2.94 (2.86–3.01)** | **2.77 (2.67–2.87)** |
|  | **0.005** | **<.001** | **<.001** | **<.001** | **<.001** | **<.001** |

Results in bold indicate statistical significance (*p* < .05)

Data are expressed as adjusted odds ratio (95% confidence interval) with *p* value (using the chi-square test). Multivariate logistic regression models controlled for maternal age, race and ethnicity, education level, nativity, demographic region, source of prenatal care payment, Federal Supplemental Nutrition Program for Women, Infants, and Children participation, first-trimester prenatal care initiation, and maternal smoking status

LBW: low birth weight; PTB: preterm birth; VLBW: very low birth weight; VPTB: very preterm birth
