# Supplementary material for: Trends in maternal prepregnancy body mass index (BMI) and its association with birth and maternal outcomes in California, 2007–2016: A retrospective cohort study
Source: PLoS One. 2019 Sep 19;14(9):e0222458. doi: 10.1371/journal.pone.0222458 (PMC6752764; doi:10.1371/journal.pone.0222458)
Supplement: S1 Checklist — (DOCX) [file pone.0222458.s005.docx]

# S1 Checklist. STROBE Statement for observational studies

|  | | Item No | Recommendation |
| --- | --- | --- | --- |
| **Title and abstract** | | 1 | 1. Indicate the study’s design with a commonly used term in the title or the abstract   **This study is a retrospective cohort study as indicated in the abstract (Pages: 3-4).** |
|  |  |  | 1. Provide in the abstract an informative and balanced summary of what was done and what was found   **The abstract contains the following sections: objectives, methods, results, and conclusions (Pages: 3-4).** |
| Introduction | | | |
| Background/rationale | | 2 | Explain the scientific background and rationale for the investigation being reported  **This is outlined in pages 7-11.** |
| Objectives | | 3 | State specific objectives, including any prespecified hypotheses  **This is outlined in page 6.** |
| Methods | | | |
| Study design | | 4 | Present key elements of study design early in the paper  **The abstract contains all the key elements of the paper.** |
| Setting | | 5 | Describe the setting, locations, and relevant dates, including periods of recruitment, exposure, follow-up, and data collection  **This information is provided in the Methods section (Pages 7-11).** |
| Participants | | 6 | (*a*) *Cohort study*—Give the eligibility criteria, and the sources and methods of selection of participants. Describe methods of follow-up  **Pages 7-11 and Figure 1.** |
|  |  |  |  |
| Variables | | 7 | Clearly define all outcomes, exposures, predictors, potential confounders, and effect modifiers. Give diagnostic criteria, if applicable  **Pages 7-11, Table 1, and Table 2.** |
| Data sources/ measurement | | 8* | For each variable of interest, give sources of data and details of methods of assessment (measurement). Describe comparability of assessment methods if there is more than one group  **Pages 7-11 and Figure 1.** |
| Bias | | 9 | Describe any efforts to address potential sources of bias  **We employed multivariate logistic regression modelling approach to control potential confounding variables. This is outlined in Table 3 and Table 4, S1 Table, S2 Table, and S3 Table.** |
| Study size | | 10 | Explain how the study size was arrived at  **Figure 1 provides details of how we arrived at the study population. This information is also provided in the Methods section (Pages 7-11).** |
| Quantitative variables | | 11 | Explain how quantitative variables were handled in the analyses. If applicable, describe which groupings were chosen and why  **Methods section (Pages 7-11).** |
| Statistical methods | |  | (*a*) Describe all statistical methods, including those used to control for confounding **The statistical methods are described on page 7-11.** |
|  |  |  | 1. Describe any methods used to examine subgroups and interactions   **Tis is described in Table 1, Table 2, Fig. 1, Fig 2, S1 Table and S2 Table.** |
|  |  |  | 1. Explain how missing data were addressed   Cases with missing data for variables within the model were excluded |
|  |  |  | 1. *Cohort study*—If applicable, explain how loss to follow-up was addressed   **No loss to follow-up.** |
|  |  |  | 1. Describe any sensitivity analyses   **Sensitivity analyses: Both observed and predicted information and crude and adjusted information were presented in tables and figures. Series of univariate and multivariate modelling approaches were used to improve sensitivity. Appropriate p values with 95% confidence intervals were provided in tables as well as figures. Study results were compared with information available in literature. Error bars were presented in figures whenever possible.** |
| Results | | | |
| Participants | 13* | 1. Report numbers of individuals at each stage of study—eg numbers potentially eligible, examined for eligibility, confirmed eligible, included in the study, completing follow-up, and analysed   **Pages 7-11 and Figure 1.** | |
|  |  | 1. Give reasons for non-participation at each stage   **N/A** | |
|  |  | 1. Consider use of a flow diagram   **A flow diagram is included Figure 1.** | |
| Descriptive data | 14* | 1. Give characteristics of study participants (eg demographic, clinical, social) and information on exposures and potential confounders   **These characteristics are included in Table 1 and 2** | |
|  |  | 1. Indicate number of participants with missing data for each variable of interest   **Figure 1, Table 1, and Table 2.** | |
|  |  | 1. *Cohort study*—Summarise follow-up time (eg, average and total amount)   **N/A** | |
| Outcome data | 15* | *Cohort study*—Report numbers of outcome events or summary measures over time  **N/A** | |
|  |  | *Case-control study—*Report numbers in each exposure category, or summary measures of exposure  **N/A** | |
|  |  | *Cross-sectional study—*Report numbers of outcome events or summary measures  **N/A** | |
| Main results | 16 | 1. Give unadjusted estimates and, if applicable, confounder-adjusted estimates and their precision (eg, 95% confidence interval). Make clear which confounders were adjusted for and why they were included   **Page 11-16.** | |
|  |  | (*b*) Report category boundaries when continuous variables were categorized  **Clearly described in methods pages 7-11.** | |
|  |  | 1. If relevant, consider translating estimates of relative risk into absolute risk for a meaningful time period   **Not relevant.** | |
| Other analyses | 17 | Report other analyses done—eg analyses of subgroups and interactions, and sensitivity analyses  **Page 11-16** | |
| Discussion | | | |
| Key results | 18 | Summarise key results with reference to study objectives  **Page 17-23** | |
| Limitations | 19 | Discuss limitations of the study, taking into account sources of potential bias or imprecision. Discuss both direction and magnitude of any potential bias  **Page 22-23** | |
| Interpretation | 20 | Give a cautious overall interpretation of results considering objectives, limitations, multiplicity of analyses, results from similar studies, and other relevant evidence  **The discussion section considers our results in light of previous findings in this domain.** | |
| Generalisability | 21 | Discuss the generalisability (external validity) of the study results  **This is described in the discussion section, last paragraph.** | |
| Other information | | | |
| Funding | 22 | Give the source of funding and the role of the funders for the present study and, if applicable, for the original study on which the present article is based  **The authors received no specific funding for this work.** | |

*Give information separately for cases and controls in case-control studies and, if applicable, for exposed and unexposed groups in cohort and cross-sectional studies.

**Note:** An Explanation and Elaboration article discusses each checklist item and gives methodological background and published examples of transparent reporting. The STROBE checklist is best used in conjunction with this article (freely available on the Web sites of PLoS Medicine at http://www.plosmedicine.org/, Annals of Internal Medicine at http://www.annals.org/, and Epidemiology at http://www.epidem.com/). Information on the STROBE Initiative is available at www.strobe-statement.org.
